# *C. elegans* VWA-8 is a mitochondrial protein

**DOI:** 10.17912/micropub.biology.000264

**Published:** 2020-06-03

**Authors:** Ming Zhu, Andrew D Chisholm, Yishi Jin

**Affiliations:** 1 Section of Neurobiology, University of California San Diego, La Jolla, CA 92093, United States

**Figure 1:**
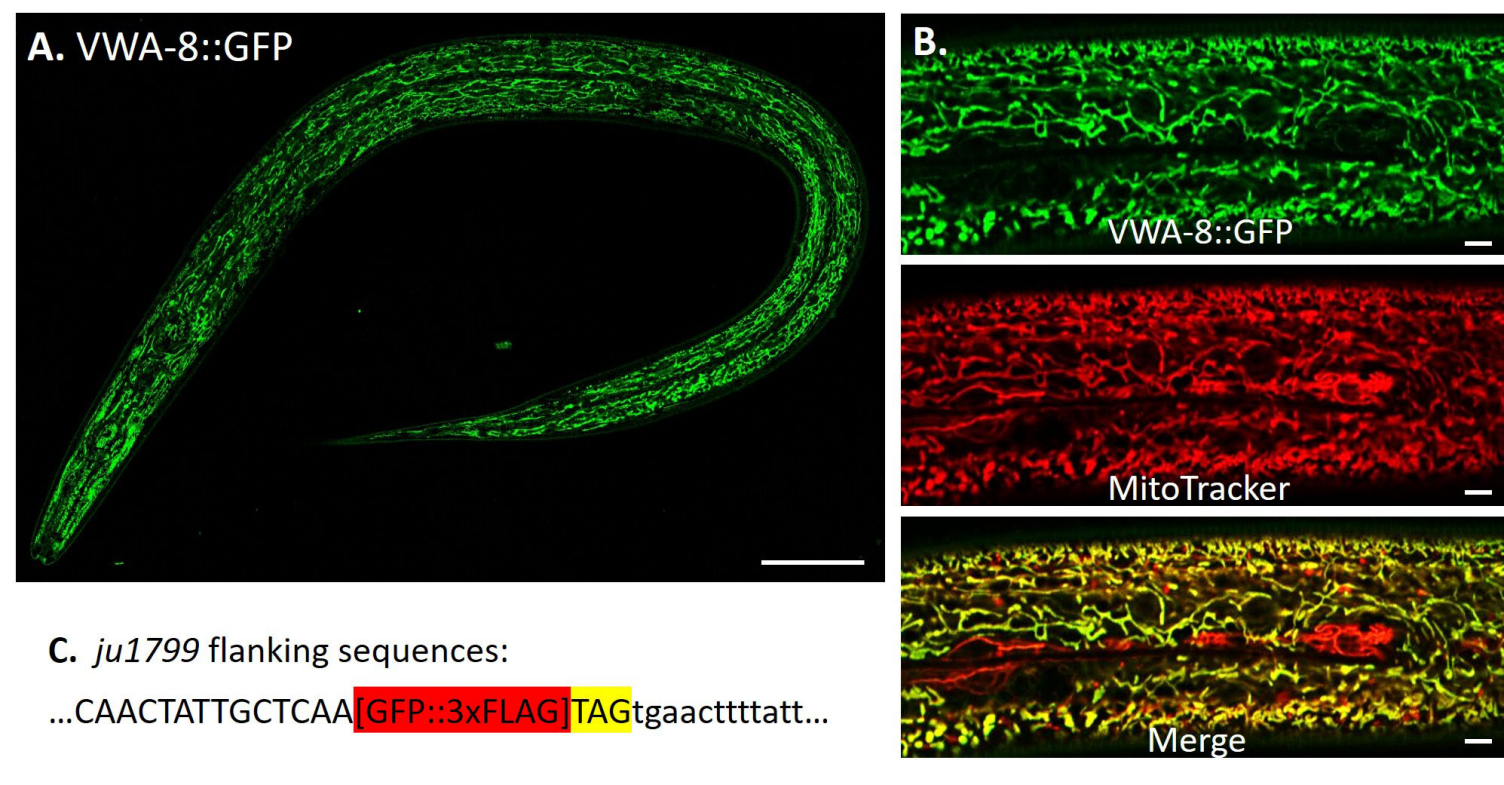
**(A)** The endogenous expression pattern of VWA-8::GFP (*ju1799)*. Scale bar: 50µm. **(B)** In hypodermis, VWA-8::GFP colocalizes with a mitochondria marker (MitoTracker Red CMXRos). Scale bar: 5µm. **(C)** The GFP::3xFLAG tag is inserted before the stop codon (highlighted in yellow) of the F11C1.5a.1 isoform. Exonal sequences are shown in uppercase.

## Description

VWA8 proteins, named for von Willebrand factor A (VWA) domain containing 8, are conserved from worm to mammals (Whittaker & Hynes, 2002). In mouse, *vwa8* gene produces long and short protein isoforms (VWA8a and VWA8b), and in rat livers VWA8a has been shown to localize to the matrix side of inner mitochondrial membrane (Luo *et al.*, 2020).

*C. elegans vwa-8* is also predicted to produce two major protein isoforms. The short isoform (974 AA) shares identical AA sequences as the long isoform (1804 AA). The two isoforms contain an MTS (mitochondrial targeting sequence) at the N terminus, followed by three AAA ATPase domains, which are associated with diverse cellular activities; and the long isoform contains a VWA domain at the C terminus.

To determine the endogenous expression pattern of *C. elegans* VWA-8, we generated a GFP knock-in allele, *ju1799,* in which GFP was in-frame fused at the C terminus to label the full-length protein specifically. The endogenous VWA-8::GFP was expressed in mitochondria of hypodermis, intestine and muscle, but was not detectable in neurons (Fig 1A). In hypodermis, VWA-8::GFP was colocalized with MitoTracker Red, a mitochondria marker (Fig 1B).

## Methods

**CRISPR-mediated GFP knock-in**: A GFP::3xFLAG tag was inserted right before the STOP codon of *vwa-8* long isoform, following a CRISPR-Cas9 protocol (Dickinson, Pani, Heppert, Higgins, & Goldstein, 2015). We designed a subgenomic RNA (sgRNA): AGCAATAGTTGATGAGAAAA targeting the stop codon of *vwa-8*. We injected 50 ng/µl of *vwa-8* sgRNA, 10 ng/µl of homology arm repair template, 2.5 ng/µl *Pmyo-2::mCherry* and 5 ng/µl of *Pmyo-3::mCherry* into wild type worms. 3 days after injection hygromycin was added to the plates to kill the untransformed F1 animals. On day 6 post-injection, we looked for candidate GFP knock-in animals which were L4/adult roller, survived hygromycin selection and without the mCherry extrachromosomal array markers. We then heat shocked 20 L1/L2 candidate knock-in worms at 34°C for 4 hours to remove the self-excising cassette. After that, the WT-looking worms were the final GFP knock-in animals. The GFP fluorescence was examined using compound microscopy. GFP insertion was confirmed by PCR using primers: 5’-GGGGCGGATGATGAGAAGTT-3’ and 5’-TGCTCTCGAACACCTTGCTT-3’.

**MitoTracker Red CMXRos staining**: Worms were soaked in 50 µl MitoTracker Red CMXRos (2.5 µM in M9 buffer; Invitrogen M7512) for 10 min at 20°C in the dark. Then worms were transferred to an OP50-seeded NGM plate and allowed to recover for 2 h at 20°C in the dark.

**Imaging**: Fluorescence images were collected using Zeiss LSM800 confocal microscopy. Worms were anesthetized with 2µM of levamisole.

## Reagents

CZ27748 *vwa-8::GFP::3xFLAG (ju1799)* will be available at the CGC.
